# Surgical Precision of MICA and Reverdin-Isham Using 3D-Printed Guides: A Cadaveric Study

**DOI:** 10.3390/medicina60101613

**Published:** 2024-10-02

**Authors:** Nadia Fernández-Ehrling, Eduardo Nieto-García, Leonor Ramirez-Andrés, Elena Nieto-González, Carlos Barrios, Sergio García-Vicente, Javier Ferrer-Torregrosa

**Affiliations:** 1 Podiatry Department, Faculty of Medicine and Health Sciences, Catholic University of Valencia San Vicente Mártir, C/Ramiro de Maeztu 14, 46900 Torrent, Spain; nadia.fernandez@ucv.es (N.F.-E.); eduardo.nieto@ucv.es (E.N.-G.); leonor.ramirez@ucv.es (L.R.-A.); elena.nieto@ucv.es (E.N.-G.); 2 Institute for Research on Musculoskeletal Disorders, Faculty of Medicine and Health Sciences, Catholic University of Valencia San Vicente Mártir, C/Quevedo 2, 46001 Valencia, Spain; carlos.barrios@ucv.es; 3 Sagunto Hospital, Generalitat Valenciana, C/Ramón y Cajal 1, 46520 Sagunto, Spain; sergiogvi@comv.es

**Keywords:** 3D printing, minimally invasive surgery, hallux valgus, surgical precision, osteotomy, patient safety, cadaver study

## Abstract

*Background/Objectives*: Hallux valgus is a common foot deformity that requires surgical correction to restore proper alignment. Precision in the osteotomies performed during these surgeries is critical to avoid complications and improve outcomes. However, variability in surgeon experience can negatively affect precision. In this context, advances in 3D printing have enabled the development of customized surgical guides, which may enhance precision and reduce variability among surgeons with different levels of expertise. This study aims to evaluate the effectiveness of a 3D-printed surgical guide in minimally invasive hallux valgus correction, focusing on the accuracy of osteotomies performed by novice surgeons, experienced surgeons, and theoretically trained consultants. *Methods*: An ex vivo study was performed with 30 cadaveric feet, divided into three groups according to the level of experience of the surgeons: 3D guide group, Master’s students, professionals. All surgeons performed Akin and Reverdin-Isham osteotomies, but the experimental group (the 3D guide group) utilized a customized 3D-printed surgical guide for enhanced precision during these procedures. Radiographic measurements of osteotomy angles and alignment were taken after the interventions, and compared with the planned values. Statistical analyses were conducted to evaluate the variability in the precision of the cuts. *Results*: The use of the 3D-printed surgical guide significantly reduced angular variability in the experienced group, achieving higher levels of accuracy than experienced surgeons. Effect sizes, which ranged from small to large, indicated a greater impact on angle measurements (η^2^ = 0.46, *p* < 0.001); no significant differences were found between the groups in other evaluated parameters. *Conclusions*: The incorporation of 3D-printed surgical guides in hallux valgus surgery significantly improves osteotomy accuracy, particularly in less experienced surgeons. This suggests that these guides can help standardize procedures, reduce the learning curve, and lower intraoperative complications.

## 1. Introduction

Minimally invasive surgery for moderate hallux abductus valgus is an increasingly used technique [1]. This surgical modality has the advantage of reducing damage to soft structures [2] compared with traditional open techniques. However, minimally invasive surgery also comes with certain challenges. One of the main challenges is the need for a high learning curve [3] for surgeons, which implies a prolonged period of training and adaptation to the specific techniques of this surgical modality.

During minimally invasive surgery, the use of continuous radiological controls by intraoperative fluoroscopy is required. This is because, due to the small incisions, the surgical field is not directly visualized, thus increasing the risk of complications [2]. Lack of adequate vision or inexperience with the techniques can lead to injury to important anatomical structures [2], underscoring the need for a high level of skill and precision on the part of the surgeon.

Despite these challenges, several studies have shown that when minimally invasive surgery is performed by experienced surgeons [1,3,4,5], it is a safe technique. Current technology, through the use of CAT scans and computerized design, allows for more precise planning of surgeries with greater accuracy. This planning, together with the acquisition of physical models by stereolithographic rapid prototyping [6], allows the fabrication of customized surgical guides [7], thus improving the accuracy of interventions [8].

In other fields, such as dentistry [7,9], the evolution in implant surgery with surgical positioners has shown significant progress, evidencing the potential of these technologies in various medical areas. The aim of this study is to introduce a new concept in minimal incision foot surgery using a standard 3D-printed surgical guide and to evaluate the accuracy of this method ex vivo.

## 2. Materials and Methods

### 2.1. Study Design

The ex vivo study was carried out using 30 normal cadaveric feet, carefully selected to ensure consistency and reliability of the results. This study was conducted according to Strobe guidelines [10].

All procedures were performed in a specialized laboratory with Xiscan 4400 Series Fluoroscopy from Fm Control/Group Alcor (Alava, Spain), which allowed detailed visualization during the procedures. The surgeons/study participants were divided into three groups according to their level of experience and familiarity with Minimal Incision Surgery (MIS).

3D guidewire group:

This group consisted of 2 consultants who had attended training courses in minimally invasive surgery (MIS) but did not routinely practice these techniques in their daily clinical work. Their participation was crucial to assess the impact of customized surgical guidelines on professionals with theoretical training but no regular practical experience in these procedures.

Master’s student group:

This group consisted of 10 surgeons with limited experience in foot surgery. These professionals were in the early stages of their surgical career, with less than 5 years of experience performing minor surgeries and few procedures to correct hallux valgus. This group represented surgeons in training, whose skills were still developing.

Professional group:

This group included 10 surgeons with more than 15 years of surgical experience, specializing in foot surgery. These highly experienced professionals had a long history of performing complex procedures and served as a reference to evaluate how experience influences the performance of the surgical techniques studied.

To determine the existence of significant differences in the cut-off measures between three groups, a sample size calculation was performed using analysis of variance (ANOVA). Considering a significance level (α) of 0.05 and a test power (1-*β*) of 0.80, a large effect size (0.40) was established. The results indicated that 8 specimens per group would be required to ensure the detection of significant differences with these parameters. This setting allows working within the experimental limits while maintaining a reasonable probability of identifying relevant differences between groups.

### 2.2. Inclusion and Exclusion Criteria

To ensure the homogeneity and relevance of the results, strict inclusion criteria were established. Only cadaveric feet without significant structural deformities and with a length between 25.4 and 25.8 cm (equivalent to sizes 41–42 EU) were selected. These criteria made it possible to standardize the study conditions and minimize the variables that could affect the results.

Feet with previous deformities or injuries that could interfere with the accuracy of the osteotomies were excluded from the study. In addition, feet that did not comply with the established length measurements were discarded in order to maintain consistency in the sample conditions.

A total of 30 feet were selected, 10 in each group were included, although 2 of the feet in the 3d guise experimental group could not be evaluated due to the incorrect positioning of the surgical guide. There was a randomization of the samples that ensured that all groups had an equal opportunity to perform the procedures without specimen selection bias.

### 2.3. Surgical Technique

Surgeons in groups 1 and 2 performed a minimally invasive Reverdin-Isham procedure along with Akin Osteotomy to correct hallux valgus. All surgeons in both groups were given precise instructions to perform the osteotomies at a distance of 16 mm from the metatarsophalangeal joint line. For group 3, composed of non-MIS surgeons, a standard 3D-printed surgical guide was designed and fabricated. This guide, made of a single piece of 2-mm thick Polyamide 12, was adapted to the foot and covered the entire medial aspect of the first toe. The guide included two holes for fixation insertion using 2 mm K-wires and a 0.2 mm radiopaque element at 16 mm from the osteotomies for precise alignment with the joint line.

In this way all participants would try to perform the Reverdin and Akin osteotomies at 16 mm from the metatarsophalangeal joint line of the first toe.

#### 2.3.1. Design Features of the Standard Surgical Guide

The standard surgical glove is 3D-printed to fit the first ray of the foot and is made from a single piece of polyamide 12, a material resistant to the high temperatures of an autoclave (125 °C–135 °C), ensuring its sterilization for intraoperative use. The material has a thickness of 2 mm, and at both cranial and caudal ends there are two holes for inserting Kirschner wires to secure the glove to the patient’s first ray. The guide covers the entire medial aspect of the first toe, extending from the proximal dialysis joint of the 1metatarsal to the most distal part of the toe, wrapping around both the dorsal and plantar sides of the first ray.

**Figure 1:**
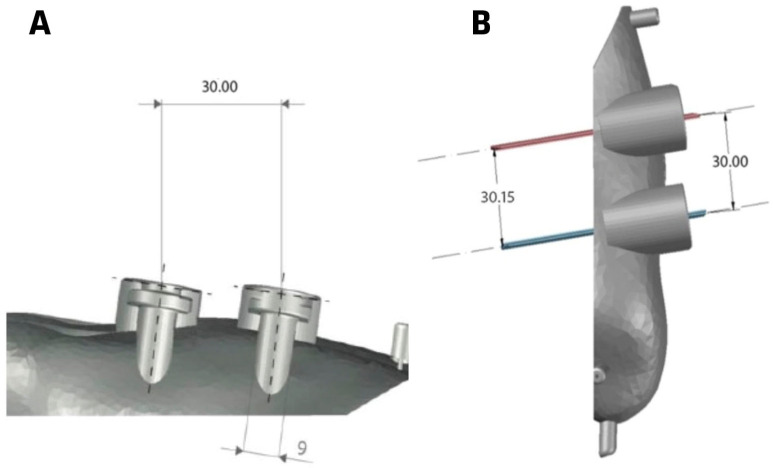
Surgical guide for group 3. (**A**) inferior vision, (**B**) superior vision.

As seen in Figure 1, the guide includes two slots where the osteotomies are projected, with the distal slot corresponding to the Akin osteotomy and the proximal one to the Reverdin osteotomy. Figure 1 shows that the distance between the guides is 3 cm, and the width of each guide slot is 9 mm.

Fluoroscopic verification will confirm if the osteotomies are properly aligned with the anatomical landmarks of the first ray, as the aluminum wire should align with the interarticular line of the first metatarsophalangeal joint. The Akin and Reverdin osteotomies must be parallel to the metatarsophalangeal joint line and located 1.6 cm away from it.

Once the guides are verified to be correctly positioned using the fluoroscope, the glove is secured to the first ray with two Kirschner wires—one inserted at the most distal part and the other at the most proximal part. After completing the evaluation, positioning, and fixation of the glove, the next step is to perform the Akin and Reverdin osteotomies.

#### 2.3.2. Operative Technique

The surgical procedure was the performance of the Reverdin-Isham technique which consists of making a precise longitudinal incision in the plantar-medial aspect of the foot, directly over the head of the first metatarsal. This incision is carefully deepened to reach the capsule of the first metatarsophalangeal joint, ensuring minimal disruption of the surrounding tissues. Once the capsule is exposed, a medium Shannon reamer is introduced through the incision. A wedge-shaped osteotomy is then performed, angled dorsally and distally toward the plantar and caudal aspects of the metaphyseal area of the metatarsal head. This angulation is crucial to achieve the desired correction while maintaining structural integrity. The technique must be executed with extreme care to preserve the lateral cortex.

After completing the osteotomy, the surgeon verifies its accuracy using a fluoroscope. Once the position is confirmed, the toe is gently rotated in abduction, which helps to compress and stabilize the Reverdin’s osteotomy site. The Akin osteotomy is then performed. This involves making an incomplete bony cut at the base of the first proximal phalanx, paying particular attention to preserving the lateral cortex. The Akin osteotomy also helps to correct the alignment of the finger.

For surgeons in the group using the 3D-printed surgical guide, the procedure was enhanced by attaching the guide to the first ray with the aid of a fluoroscope. The radiopaque line embedded in the guide was aligned with the joint line to ensure correct placement. The guide was then firmly attached to the first ray using two K-wires, one placed proximally and the other distally, providing stability for both the Reverdin and Akin osteotomies.

### 2.4. Radiographic Measurements

**Figure 2:**
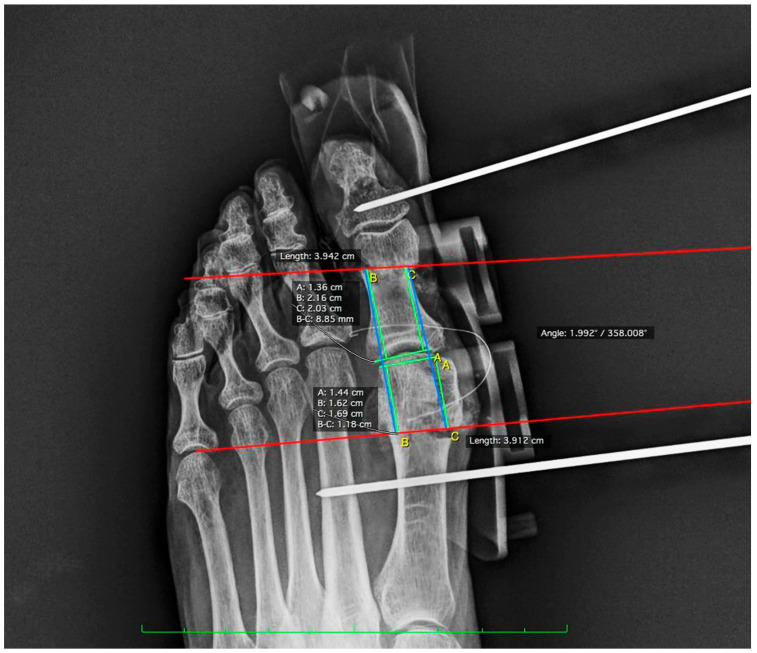
Post-surgical radiographic measurements lines using the Osirix program.

To evaluate the results, post-operative radiographs were taken with simulated loading. The images obtained were analyzed with Osirix MD Software (Pixmeo, Bernex, Switzerland). In measurement 1, the distance from the medial proximal end of the Akin osteotomy to the base of the proximal phalanx at the joint line was measured. This parameter is key to assessing the accuracy of the osteotomy alignment with respect to the surrounding articular structures. In measurement 2, the distance from the proximal lateral end of the Akin osteotomy to the base of the proximal phalanx at the joint line was determined. This measurement complements the first measurement by providing a complete perspective of the osteotomy orientation. Measurement 3 consisted of calculating the distance from the medial distal end of the Reverdin osteotomy to the articular interline formed by the head of the first metatarsal. In measurement 4, the distance from the distal lateral end of the Reverdin osteotomy to the articular interline formed by the head of the first metatarsal and the base of the proximal phalanx was measured to verify symmetry and correct placement of the osteotomy on the lateral side. Finally, angle measurement evaluated the angle formed by the two previous osteotomies (see Figure 2).

### 2.5. Statistical Analysis

All analyses were performed by an observer outside the experimental conditions. Data were expressed as mean and standard deviation (SD). Normality was assessed using the Shapiro–Wilk test. In addition, homogeneity of variance was calculated using Levene’s test. The significance level was set at *p* < 0.05. SPSS 24 (SPSS 24 Inc., Chicago, IL, USA) and Jeffreys’s Amazing Statistical Package (JASP)(0.18.3, Amsterdam, The Netherlands) were used to perform statistical treatment and the graphical representation of the data, respectively.

Analyses of variance (ANOVA) were performed to compare the means of several measures (Measure1 to Measure4, and Angle) between three different groups: 3D Guide, Master’s Students, and Professionals. In addition, effect sizes were calculated using eta (η^2^) and omega squared (*ω*^2^), and we classified them into small effect: *ω*^2^ ≈ 0.01; moderate: *ω*^2^ ≈ 0.06; and large: *ω*^2^ ≈ 0.14 to assess the magnitude of differences between groups [11,12].

All analyses were performed using statistical analysis software (JASP, the Netherlands). The level of statistical significance was set at *p* < 0.05, implying that a statistically significant association would be considered when the *p* value was less than 0.05.

## 3. Results

The study evaluated various measures (M1 to M4 and Angle) in three groups: 3D Guide, Professionals, and Master’s Students. The results are presented in a comparative table that includes descriptive statistics for each group, such as the number of samples (No.), mean, standard deviation (SD), and minimum and maximum values. In addition, the Table provides statistical significance values (*p*-values) and effect size measures (η^2^ and *ω*^2^), which show the strength of the associations in the different metrics evaluated (Table 1).

For the measures Measure1 and Measure2, the 3D Guide group showed means of 15.10 (SD = 2.76) and 16.10 (SD = 2.83), respectively. The Master’s group obtained means of 12.03 (SD = 3.94) and 12.59 (SD = 3.67), while the Professionals group recorded means of 13.25 (SD = 2.62) and 13.56 (SD = 5.00). Although ANOVA analyses did not show statistically significant differences in either measure (Measure1: F = 2.05, *p* = 0.15; Measure2: F = 1.77, *p* = 0.19), the effect size in both cases was moderate (Measure1: η^2^ = 0.14, *ω*^2^ = 0.07; Measure2: η^2^ = 0.12, *ω*^2^ = 0.05), indicating that there were appreciable, though inconclusive, differences between the groups.

For Measure3 and Measure4, the differences between the groups were minimal. For Measure3, the 3D Guide group had a mean of 17.01 (SD = 2.78), while the Master’s and Professional groups had means of 15.45 (SD = 5.74) and 17.06 (SD = 5.57), respectively. ANOVA indicated F = 0.32 (*p* = 0.73) with a very small effect size (η^2^ = 0.02, *ω*^2^ = 0.00). Similarly, in Measure4, the means were 16.90 (SD = 3.30) for 3D Guide, 15.69 (SD = 6.53) for Master’s, and 15.68 (SD = 6.86) for Professionals, with ANOVA showing F = 0.12 (*p* = 0.89) and an equally small effect size (η^2^ = 0.01, *ω*^2^ = 0.00), suggesting high similarity between the groups in both measures.

Finally, for the angle measure, the 3D Guide group obtained a mean of 2.73 (SD = 0.36), while the Master’s and Professionals groups recorded much higher means: 14.27 (SD = 4.68) and 14.84 (SD = 9.07), respectively. ANOVA revealed a statistically significant difference (F = 10.66, *p* < 0.001), with a large effect size (η^2^ = 0.46, *ω*^2^ = 0.42), suggesting substantial and significant differences between the groups in this measure.

Although most of the measures did not show statistically significant differences, some (Measure1, Measure2) exhibited moderate effect sizes, indicating appreciable differences. However, the angle measure stood out due to its statistical significance and considerable effect size, suggesting clear and significant differences between the groups.

**Figure 3:**
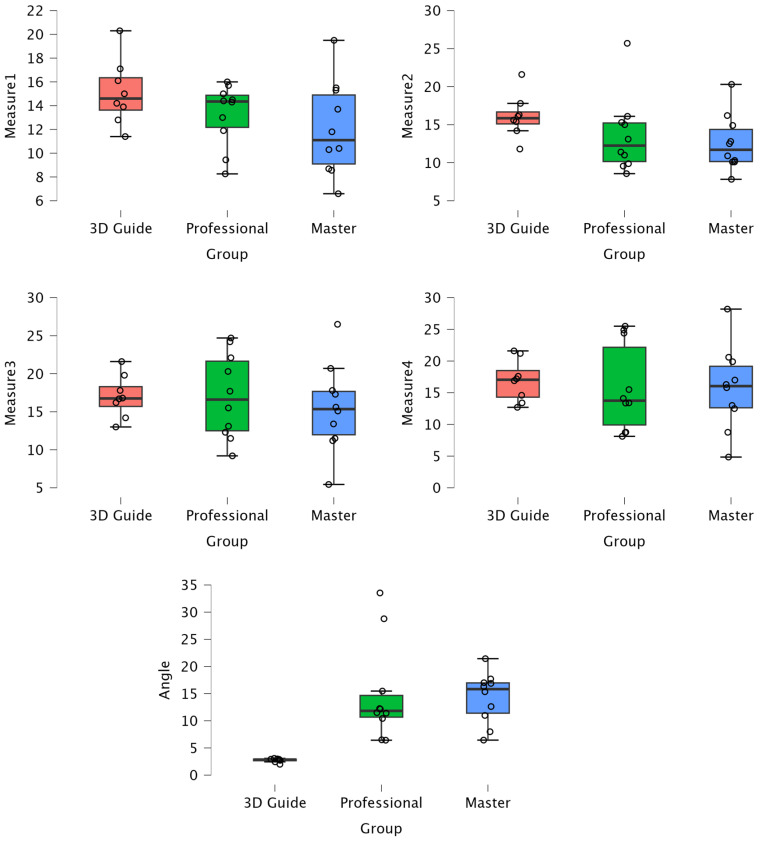
Comparison of mean values and dispersion of the 3D Guide, Master’s, and Professionals Groups for all measures. The figure displays the median, interquartile range, and outliers for each group.

In Figure 3, we observe box plots for each variable studied in each group. We can see that the 3D Guide group consistently shows much less dispersion compared to the other groups. This suggests that the 3D Guide standardizes the results across the different surgeries performed, closely aligning with the planned variable.

It can be observed that the 3D Guide group consistently exhibits lower dispersion across all measures, indicating greater consistency in its results. In contrast, the Professionals and Master’s students groups show greater variability, particularly in the Angle measure, where more pronounced differences between the groups are evident.

## 4. Discussion

The results of this study have clearly demonstrated that the use of 3D-printed surgical guides can significantly improve the precision of osteotomies in minimally invasive hallux valgus surgery. This study strongly validates the hypothesis that surgical guides not only reduce variability but also enhance accuracy in procedures performed by surgeons with varying levels of experience. The importance of these findings lies in the potential to standardize complex procedures, providing an additional tool to improve surgical outcomes.

One of the most remarkable results of this study is that the 3D-printed guides allowed the surgeons in this group to achieve a very high level of accuracy with minimal within-group variability. This is especially relevant given that the learning curve for minimally invasive techniques is notoriously long, as documented in previous studies [13,14,15]. The reduction in variability of osteotomies observed in the group that used the printed guides suggests that these tools can standardize procedures that would otherwise heavily rely on the individual surgeon’s skill [13,16].

These guides improve measured and planned outcomes in the same way that has already been observed in other fields such as dentistry [9,17,18,19] or traumatology [20,21]. This standardization capability could be a decisive factor in improving surgical outcomes and reducing post-operative complication rates and radiation exposure [16].

In terms of accuracy, the data indicate that the deviation in osteotomies across the groups was notably smaller when the surgical guide was used. This level of precision is crucial to prevent post-operative complications, reduce the number of complications in novice surgeons’ initial surgeries, as highlighted by Matjaž Merc’s study [16], and ensure the proper correction of hallux valgus. Additionally, the use of standard surgical guides allows for more precise pre-operative planning, helping to avoid potential human errors and post-operative complications, such as overcorrections. This aspect is critical to ensuring patient safety and the effectiveness of the procedure.

The surgical guide designed for this study not only provided a physical reference for the location of the osteotomies but also included radiopaque elements that allowed for the precise intraoperative verification through fluoroscopy [13,16,22,23], while reducing radiation exposure during surgery, as the fluoroscope is only used during the placement of the guide. The surgical guide was created for feet of a specific size and with particular characteristics. This standardized design of the guide represents a significant advance, since it allows the procedure to be adapted to the needs of the patients, which is essential to improve surgical results, without the need for the exhaustive individualization of the guide.

With these standard surgical guides, the angulation of the osteotomies can be standardized, and it can be ensured that the osteotomies are performed exactly in the planned location [24], as the distance from the radiopaque element is aligned with the joint. This millimetric precision is crucial for achieving the best possible outcomes and minimizing the risk of complications [15,16].

The study does present several limitations and biases. First, the use of cadaveric feet may not fully reflect real surgical conditions, as they lack the dynamic factors present in living patients, such as biological responses to surgery and healing. Additionally, the sample size is limited, with only 28 cadaveric feet, which may affect the generalization of the results. There may also be variability in the skill level of novice surgeons, which could influence the results, although the use of surgical guides aims to minimize this factor. Lastly, the exclusion of feet with deformities may limit the applicability of the study to more complex cases.

## 5. Conclusions

The integration of 3D-printed surgical guides into minimally invasive hallux valgus surgery represents a significant advancement. These guides not only help standardize procedures and improve precision, but they can also accelerate the learning curve for novice surgeons, reduce surgical time, and minimize radiation exposure, thus improving both patient and professional safety. Future studies should focus on further optimizing guide designs and evaluating their effectiveness in a broader clinical context.

## 6. Patents

Drs. Ferrer-Torregrosa and Garcia-Vicente are inventors of the guiding patent and holding device for minimum-incision foot surgery (2014 PCT/ES2013/070877).

## Figures and Tables

**Table 1:** Comparison of descriptive statistics and significance analysis between groups for the variables studied.

Group		M1	M2	M3	M4	Angle
3D Guide						
	No.	8	8	8	8	8
	Mean	15.10	16.10	17.01	16.90	2.73
	S.D	2.76	2.83	2.78	3.30	0.36
	Minimum	11.40	11.80	13.00	12.70	1.99
	Maximum	20.30	21.60	21.60	21.60	3.08
Master’s students						
	No.	10	10	10	10	10
	Mean	12.03	12.59	15.45	15.69	14.27
	S.D	3.94	3.67	5.74	6.53	4.68
	Minimum	6.59	7.82	5.44	4.85	6.44
	Maximum	19.50	20.30	26.50	28.20	21.43
Professionals						
	No.	10	10	10	10	10
	Mean	13.25	13.56	17.06	15.68	14.84
	S.D	2.62	5.00	5.57	6.86	9.07
	Minimum	8.26	8.56	9.20	8.12	6.42
	Maximum	16.00	25.70	24.70	25.50	33.53
Statistics	*p*-value	0.15	0.19	0.73	0.89	<0.001 ^a^
	η^2^	0.14	0.12	0.02	0.01	0.46
	*ω* ^2^	0.07	0.05	0.00	0.00	0.41

Note: see the ‘‘Section 2.2’’ under ‘‘Section 2’’ for definitions of the seven measurements. Abbreviations: M, measure; S.D, standard deviation. ^a^ Kruskal–Wallis test.

## Data Availability

The data presented in this study are available upon request at https://data.mendeley.com/datasets/4ygx6shnhz/1 (accessed on 28 August 2024).
